# Hydroxypyruvate Reductase 1 and Plastidial Glycolate/Glycerate Transporter 1 Connect Photorespiration With Plant Immunity

**DOI:** 10.1111/pce.70639

**Published:** 2026-06-01

**Authors:** Xiaotong Jiang, Sheng Yang He, Jianping Hu

**Affiliations:** ^1^ Department of Energy Plant Research Laboratory Michigan State University East Lansing Michigan USA; ^2^ Current address: Department of Biology Pennsylvania State University University Park Pennsylvania USA; ^3^ Howard Hughes Medical Institute and Department of Biology Duke University Durham North Carolina USA; ^4^ Department of Plant Biology Michigan State University East Lansing Michigan USA

## Abstract

By a combination of genetic, transcriptomic and pathogen infection analyses, this study shows a connection between two *Arabidopsis* photorespiratory proteins, HPR1 and PLGG1, and plant immunity. Elevated CO_2_ largely rescues the altered susceptibility of the *hpr1* and *plgg1* mutants to bacterial infection, highlighting the potential role of photorespiration in effective immune response in plants.

Plants have developed a sophisticated immune system that includes two interacting and connected layers: pattern‐triggered immunity (PTI) and effector‐triggered immunity (ETI), which contain key signalling events such as transcriptional reprogramming, reactive oxygen species (ROS) burst and biosynthesis of the phytohormones salicylic acid (SA) and jasmonate (JA) (Figure S1) (Dodds and Rathjen 2010). Other biological and metabolic pathways, such as photorespiration, are also involved and regulated during plant‐pathogen interaction. Closely linked to photosynthesis, photorespiration is initiated following the oxygenation of ribulose 1,5‐bisphosphate (RuBP) catalysed by RuBP carboxylase‐oxygenase. In photorespiration, 2‐phosphoglycolate produced by RuBP oxygenation, which inhibits cellular functions, is converted to 3‐phosphoglycerate that is recycled back to the Calvin‐Benson cycle (Figure S1) (Eisenhut et al. 2019). Evidence for the role of photorespiration in plant immunity is emerging (Jiang et al. 2023). Since photorespiration is considered a major H_2_O_2_ source in photosynthetic cells (Foyer et al. 2009), most studies have focused on ROS‐related mechanisms, while ROS‐independent mechanisms have also been reported (Jiang et al. 2023). These discoveries were obtained from various plant systems against disparate pathogens under different conditions; thus, a consensus model was hard to obtain. Additionally, very few of the previous reports consider the immune function of the pertinent photorespiratory components in the context of photorespiration, in particular, dependence on CO_2_ levels.

To further investigate the interaction between photorespiration and plant immunity, we leveraged photorespiratory mutants of the model plant *Arabidopsis thaliana* and assessed their disease phenotypes in response to bacterial pathogen *Pseudomonas syringae* pv. *tomato* strain DC3000 (*Pst* DC3000). The knockout mutants of hydroxypyruvate reductase 1 (HPR1), an enzyme that reduces hydroxypyruvate to glycerate, and plastidial glycolate/glycerate transporter 1 (PLGG1), a transporter of the photorespiratory intermediates glycolate and glycerate, consistently showed increased disease susceptibility. As previously reported (Timm et al. 2008; Yang et al. 2012), both air‐grown *hpr1‐1* and *plgg1‐1* plants, in which photorespiration is impaired but not totally abolished, exhibited smaller rosettes; *plgg1* also had leaf lesions (Figure S2a). We first tested the mutants for PTI response, using flg22 protection assay in which PTI was induced by pretreatment of 100 nM of flg22, a peptide from the conserved domain of the bacterial flagellin (Yu et al. 2017), followed by *Pst* DC3000 infiltration. Flg22‐pretreated *hpr1‐1* and *plgg1‐1* mutants showed significantly higher levels of bacterial populations and more obvious water‐soaking symptoms than Col‐0 (wild type), phenotypes that were more pronounced than those in the mock‐pretreated group (Figure 1a,b). To induce ETI, plants were inoculated with *Pst* DC3000 (*avrRpt2*), an avirulent strain carrying the effector protein AvrRpt2. AvrRpt2‐induced ETI requires the activation of the receptor protein RPS2 (Dodds and Rathjen 2010), whose null mutant *rps2‐101c* exhibited severe disease symptoms with high levels of bacterial growth, necrosis and chlorosis in leaves (Figure 1c,d). The disease symptoms were weaker in *hpr1‐1* and *plgg1‐1* than those in *rps2‐101c* but apparently stronger than in Col‐0 (Figure 1c,d). These data together indicate that both PTI and ETI are compromised in *hpr1‐1* and *plgg1‐1*.

**Figure 1:**
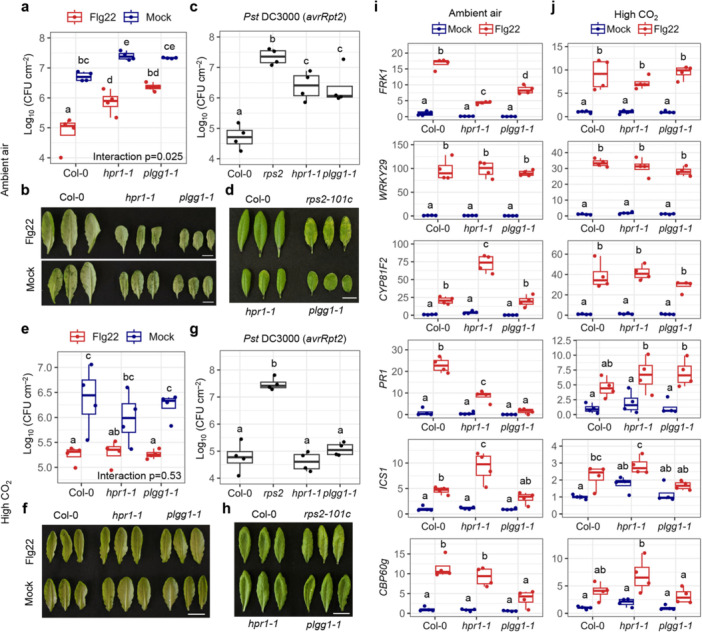
Photorespiratory mutants *hpr1* and *plgg1* show defects in immune response that can be largely rescued by high CO_2_ conditions (2000 ppm). (a, b) PTI response assays showing quantification of bacterial populations inside the leaves (a) and water‐soaking symptoms on the leaves (b) that were pretreated with 100 nM flg22 followed by infiltration 24 h later with *Pseudomonas syringae* pv. *tomato* DC3000 (*Pst* DC3000) at ~1 × 10^6^ CFU mL^−1^. Plant leaves were sampled at 2 days postinfiltration (2 dpi). Scale bar = 1 cm. (c, d) ETI response assays showing the quantification of bacterial populations inside the plant leaves (c) and disease symptoms on the leaves (d) at 3 dpi with *Pst* DC3000 (*avrRpt2*) at ~1 × 10^6^ CFU mL^−1^. Scale bar = 1 cm. (e, f) PTI response assays showing quantification of bacterial population inside the leaves (e) and water‐soaking symptoms on the leaves (f) that were pretreated with 100 nM flg22 followed by infiltration 24 h later with *Pst* DC3000 at ~1 × 10^6^ CFU mL^−1^. Leaves were sampled at 2 dpi. Scale bar = 2 cm. (g, h) Quantification of bacterial populations inside the leaves (g) and disease symptoms on the leaves (h) at 3 dpi with *Pst* DC3000 (*avrRpt2*) at ~1 × 10^6^ CFU ml^−1^. Scale bar = 2 cm. (i, j) RT‐PCR analysis of immunity marker genes. Expressions of genes in air‐grown (i) or high CO_2_‐grown (j) plants induced by 100 nM flg22 were analysed at 2 hpi for *FKR1*, *WRKY29* and *CYP81F2* and at 24 hpi for *PR1*, *ICS1* and *CBP60g*. *PP2AA3* was used as the internal control, and data were normalised to the average gene expression in mock‐treated Col‐0. Five‐week‐old plants grown under air (a‐d, i) or high CO_2_ (e‐h, j) conditions were used in the experiments. 0.1% DMSO was used as a mock. Different letters indicate statistically significant differences (*p* < 0.05), which were determined by Two‐way (a, e, i, j) or One‐way (c, g) ANOVA with Tukey's HSD test. Interactions between genotype and treatment were tested by Two‐way ANOVA; *p*‐values are labelled (a, e). Biological replicates: *n* = 4.

To determine whether the phenotypes of *hpr1* and *plgg1* in immunity are specifically linked to photorespiration, mutants were grown under high CO_2_ (2000 ppm) to inhibit photorespiration. As expected, *hpr1‐1* and *plgg1‐1* had similar morphologies to Col‐0 (Figure S2b). *Pst* DC3000 and *Pst* DC3000 (*avrRpt2*) grown on plates under ambient air or high CO_2_ had comparable growth (Figure S2c), excluding a direct influence of high CO_2_ on pathogen growth. For flg22 protection assay, *hpr1‐1* and *plgg1‐1* exhibited comparable bacterial growth and water‐soaking symptoms as Col‐0 in both flg22‐ and mock‐pretreated groups (Figure 1e,f). For ETI response, *rps2‐101c* grown under high CO_2_ still showed susceptibility to *Pst* DC3000 (*avrRpt2*), whereas the responses in *hpr1‐1* and *plgg1‐1* were similar to those in Col‐0 (Figure 1g,h). The rescue of the compromised immune response in *hpr1* and *plgg1* by high CO_2_ suggested that the effects of HPR1 and PLGG1 in immunity are overlapping and likely dependent on photorespiration.

We then used quantitative reverse transcription‐polymerase chain reaction (qRT‐PCR) to check the expression of six flg22‐induced genes. In air‐grown plants 2 or 24 hpi (hours postinfiltration) of flg22, both *hpr1‐1* and *plgg1‐1* showed reduced induction of *flg22‐induced receptor‐like kinase 1* (*FRK1*) and *pathogenesis‐related 1* (*PR1*) and similar expression of *WRKY29* compared with Col‐0, while having differential changes in the expression of the three other genes between the two mutants (Figure 1i). These results indicated that the effects of HPR1 and PLGG1 on plant immunity may overlap, but their distinct functions in the photorespiratory pathway may cause some differences in gene expression. Under high CO_2_, the altered expression of these genes was mostly reverted in the mutants, without any significant differences observed in Col‐0 (Figure 1j), reinforcing the view that HPR1 and PLGG1 likely exert their function in plant immunity largely through photorespiration.

Finally, we performed RNA‐seq to explore the effects of HPR1 and PLGG1 on immunity at the transcriptome level, using air‐grown plants infiltrated with mock or flg22 and sampled at 2 hpi. Principal component analysis showed that all four biological replicates for each genotype under mock or flg22 treatment clustered distinctly without overlaps (Figure S3a), validating the high quality of the dataset. Analysis of differentially expressed genes (DEGs) in the mock‐treated group revealed 2547 and 3873 DEGs between *hpr1‐1* and Col‐0, and *plgg1‐1* and Col‐0, respectively (Figure S3b; Data S1 and S2), among which 1082 genes were downregulated, and 550 were upregulated in both mutants (Figure S3c). In the flg22‐treated group, we identified 2800 and 2401 DEGs between *hpr1‐1* and Col‐0, and *plgg1‐1* and Col‐0, respectively (Figure S3d; Data S3 and S4), among which 495 downregulated and 555 upregulated genes are shared by the mutants (Figure S3e). Gene Ontology enrichment analysis of these shared DEGs showed that the downregulated DEGs in both mock‐ and flg22‐treated groups are associated with immunity and defence (Figure S4a,b), indicating that both the basal and the induced expression of immunity‐ and defence‐related genes is compromised in *hpr1* and *plgg1* mutants. However, the upregulated DEGs in the two treatment groups belong to distinct functional categories. Cell cycle and cytoskeleton‐related processes are enriched in mock‐treated group (Figure S5a), suggesting a negative role of HPR1 and PLGG1 in the expression of these genes not directly linked to immunity. By contrast, photosynthesis‐related genes are enriched in the flg22‐treated group (Figure S5b), indicating a connection between photosynthesis and immunity during defence response.

We further generated heatmaps of gene expression using a subset of immunity‐related genes, including general immunity makers and SA‐ and JA‐related genes (Figure S6). Expression patterns of the six genes tested earlier by qRT‐PCR (Figure 1i) largely agreed (Figure S6). Overall, both *hpr1‐1* and *plgg1‐1* showed reduced basal expression of most immunity markers and SA‐related genes compared with Col‐0, a trend maintained to various degrees following flg22 treatment (Figure S6a,b). This data supports the overlapping as well as distinct contributions of HPR1 and PLGG1 to immunity and is consistent with the enhanced susceptibility of these mutants to *Pst* DC3000. The expression of most JA‐related genes increased in the mutants (Figure S6c), consistent with the antagonism between SA and JA in immunity (Pieterse et al. 2012). Lastly, we generated heatmaps of gene expression for photorespiration and photosynthesis. Consistent with previous publications (Zabala et al. 2015), most of these genes were suppressed by flg22 in Col‐0 (Figure S7), among which many displayed varied degrees of elevated expression in *hpr1‐1* and *plgg1‐1* under both mock and flg22 conditions (Figure S7), supporting a potential link among photorespiration, photosynthesis and immunity.

In this study, we have shown a connection between two photorespiratory components and plant immunity (Figure S1). The fact that elevated CO_2_ largely rescued the enhanced susceptibility of *hpr1* and *plgg1* to bacterial infection highlights the potential role of photorespiration in immunity. Our qRT‐PCR and RNA‐seq data also provided transcriptomic support for the contribution of HPR1 and PLGG1 to immunity and revealed coordinated changes in genes involved in immunity‐ and photosynthesis‐related pathways. However, many questions remain to be addressed. Although our data support the involvement of photorespiration in plant immunity, the signalling cascade that leads to the response and whether photorespiratory metabolites serve as signals are unknown. Meanwhile, we cannot exclude the possibility that nonphotorespiratory factors are also involved and that the immunity phenotypes in the mutants are, at least partially, resulted from their growth defects. Additionally, our preliminary study of the apoplastic ROS burst, basal intracellular ROS levels and expression of the peroxisomal H_2_O_2_ marker gene *glutathione S‐transferase TAU 24* (Queval et al. 2007) did not reveal decreased ROS levels in the mutants compared to Col‐0 (Figure S8). Whether ROS are signals in this response needs to be elucidated using more sensitive and subcellular location‐specific ROS sensors. Lastly, the decreased expression of immunity‐related genes correlated with the increased photosynthesis‐related genes in both mutants in this study, prompting our prediction that the elevated energy cost in photosynthesis limits the energy available for immune response (Figure S1). Further investigations are needed to paint a clearer view of the interplay between photorespiration and immunity and the underlying mechanisms, which may aid in the development of crop varieties with enhanced disease resistance.

## Conflicts of Interest

The authors declare no conflicts of interest.

## Supporting information


**Figure S1:** The photorespiratory pathway and the potential connection of the photorespiratory proteins HPR1 and PLGG1 with immunity.
**Figure S2:** Null mutants of *HPR1* and *PLGG1* grown in ambient air show defects in growth and pathogen response, which can be rescued by high CO_2_ (2000 ppm).
**Figure S3:** RNA‐seq analysis revealed differentially expressed genes (DEGs) in *hpr1‐1* and *plgg1‐1* compared with Col‐0.
**Figure S4:** GO term enrichment analyses of shared downregulated DEGs in both *hpr1‐1* and *plgg1‐1* mutants with mock (a) or flg22 (b) treatment.
**Figure S5:** GO term enrichment analyses of shared upregulated DEGs in *hpr1‐1* and *plgg1‐1* mutants with mock (a) or flg22 (b) treatment.
**Figure S6:** Heatmaps showing the expression of general marker genes in immunity (a), salicylic acid (SA) biosynthesis and signalling genes (b), and genes involved in jasmonate (JA) metabolism and signalling (c).
**Figure S7:** Heatmaps showing the expression of genes involved in photorespiration (a) and photosynthesis (b) in *hpr1* and *plgg1* at the basal level and in response to flg22.
**Figure S8:** Detection of reactive oxygen species (ROS) levels and a marker gene in five‐week‐old plants grown under ambient air conditions.
**Table S1:** Primers used in qRT‐PCR.

**Data S1:** List of Differentially Expressed Genes (DEGs) in mock‐treated *hpr1‐1* vs. mock‐treated Col‐0.

**Data S2:** List of Differentially Expressed Genes (DEGs) in mock‐treated *plgg1‐1* vs. mock‐treated Col‐0.

**Data S3:** List of Differentially Expressed Genes (DEGs) in flg22‐treated *hpr1‐1* vs. flg22‐treated Col‐0.

**Data S4:** List of Differentially Expressed Genes (DEGs) in flg22‐treated *plgg1‐1* vs. flg22‐treated Col‐0.

## Data Availability

RNA‐seq data have been deposited in the NCBI SRA under project number PRJNA1372828. The data that support the findings of this study are available in the Supporting Information of this article.
