# Supplementary material for: Hydroxypyruvate Reductase 1 and Plastidial Glycolate/Glycerate Transporter 1 Connect Photorespiration With Plant Immunity
Source: Plant Cell Environ. 2026 Jun 1;49(9):6554–7. doi: 10.1111/pce.70639 (PMC13436475; doi:10.1111/pce.70639)
Supplement: Supplementary file 1 [file PCE-49-6554-s001.docx]

**Figure S1.** The photorespiratory pathway and the potential connection of the photorespiratory proteins HPR1 and PLGG1 with immunity.

During plant interaction with pathogen, receptors on the plasma membrane recognize the conserved peptide from bacterial flagellin, flg22, and induce pattern-triggered immunity (PTI). Pathogens release effectors such as AvrRpt2 into the plant cell to suppress PTI response, which can be recognized by the intracellular receptor RPS2 and induce effector-triggered immunity (ETI). Both PTI and ETI consist of key components such as salicylic acid (SA), jasmonate (JA), and reactive oxygen species (ROS). Photorespiration is closely linked to photosynthesis and involves a series of reactions in the chloroplast, peroxisome, mitochondrion, and cytosol. Our previous publication provides evidence of a cytosolic shunt that complements photorespiration (Jiang *et al.*, 2025), which is indicated by the arrows in the cytosol on the left. Our data indicates that photorespiratory proteins HPR1 (hydroxypyruvate reductase 1) and PLGG1 (plastidial glycolate/glycerate transporter 1) can affect PTI and ETI, as well as immune responses such as transcriptional reprogramming, SA biosynthesis and signaling, JA biosynthesis, catabolism and signaling. ROS are unlikely the major players in HPR1 and PLGG1-mediated immunity, and more investigations are needed for other immune responses. Our results indicate that photorespiration may influence the energy allocation between photosynthesis and immunity and therefore affect immune response.

Abbreviations: 2-OG, 2-oxoglutarate; 2-PG, 2-phosphoglycolate; 3-PGA, 3-phosphoglycerate; BASS6, bile acid sodium symporter 6; CAT, catalase; GDC, glycine decarboxylase complex; GGAT, glutamate:glyoxylate aminotransferase; GLYK, glycerate kinase; GLYR1, glyoxylate reductase 1; GOX, glycolate oxidase; PGLP, 2-PG phosphatase; Rubisco, RuBP carboxylase/oxygenase; RuBP, ribulose-1,5-bisphosphate; SGAT, serine:glyoxylate aminotransferase; SHMT, serine hydroxymethyltransferase. In the chloroplast, processes in photosynthesis are labeled green.

**Figure S2.** Null mutants of *HPR1* and *PLGG1* grown in ambient air show defects in growth and pathogen response, which can be rescued by high CO_2_ (2,000 ppm).

(a, b) Five-week-old plants grown under ambient air (a) or high CO_2_ (b). White arrows indicate yellow lesions in the older leaves of *plgg1-1*. Scale bars = 5 cm.

(c) *Pst* DC3000 and *Pst* DC3000 (*avrRpt2*) grown in ambient air or high CO_2_ chambers for 4 and 3 days, respectively.

**Figure S3.** RNA-seq analysis revealed differentially expressed genes (DEGs) in *hpr1-1* and *plgg1-1* compared with Col-0.

(a) Principal Component Analysis plot of RNA-seq samples. Five-week-old plants grown under ambient air were infiltrated by 100 nM flg22 or mock (0.1% DMSO). Leaf tissue was sampled at 2 hpi. Biological replicates: n=4.

(b) Volcano plots displaying differentially expressed genes (DEGs) in two pairwise comparisons: *hpr1-1* with mock treatment vs. Col-0 with mock treatment, and *plgg1-1* with mock treatment vs. Col-0 with mock treatment.

(c) Venn diagram of DEGs in the mutants showing the 550 upregulated and 1,082 downregulated genes shared by *hpr1-1* and *plgg1-1* with mock treatment.

(d) Volcano plots displaying differentially expressed genes (DEGs) in two pairwise comparisons: *hpr1-1* with flg22 treatment vs. Col-0 with flg22 treatment, and *plgg1-1* with flg22 treatment vs. Col-0 with flg22 treatment.

(e) Venn diagram of DEGs in the mutants showing that 555 upregulated genes and 495 downregulated genes were shared by *hpr1-1* and *plgg1-1* with flg22 treatment.

In (b) and (d), genes with an absolute log2 fold ratio>1 and an adjusted p-value (padj) of <0.05 (represented logarithmically on the y-axis) are considered DEGs. The padj-value was determined by Wald test with multi-test correction using the Benjamini and Hochberg method. Blue, red, and gray dots represent the downregulated, upregulated, and the unchanged genes, respectively.

**Figure S4.** GO term enrichment analyses of shared downregulated DEGs in both *hpr1-1* and *plgg1-1* mutants with mock (a) or flg22 (b) treatment.

Top 15 enriched GO terms, ranked by Fold Enrichment, are displayed. FDR < 0.05 was used as the cut-off.

**Figure S5.** GO term enrichment analyses of shared upregulated DEGs in *hpr1-1* and *plgg1-1* mutants with mock (a) or flg22 (b) treatment,

Top 15 enriched GO terms, ranked by Fold Enrichment, are displayed. FDR < 0.05 was used as the cut-off.

**Figure S6.** Heatmaps showing the expression of general marker genes in immunity (a), salicylic acid (SA) biosynthesis and signaling genes (b), and genes involved in jasmonate (JA) metabolism and signaling (c).

Averaged gene expression from four biological replicates was used and standardized as z‑scores, which were indicated by the color legend.

**Figure S7.** Heatmaps showing the expression of genes involved in photorespiration (a) and photosynthesis (b) in *hpr1* and *plgg1* at the basal level and in response to flg22.

Averaged gene expression from four biological replicates was used and standardized as z‑scores, which were indicated by the color legend.

**Figure S8.** Detection of reactive oxygen species (ROS) levels and a marker gene in five-week-old plants grown under ambient air conditions.

(a) Apoplastic ROS burst induced by 100 nM flg22 or mock (0.1% DMSO). The upper right inset shows the mock signals at an expanded Y-axis scale for better visibility, while the X-axis keeps the same scale. Results represent the mean ± SD. Differences between mutants and Col-0 were tested by Student’s *t-test* at each time point (*p<0.05). Biological replicates: n=6 for mock-treated plants, and n=5 for flg22-treated plants.

(b) Basal level of intracellular ROS detected by H_2_DCFDA staining. Representative images showed ROS fluorescence (green), chlorophyll autofluorescence (red) and brightfield in the mesophyll of the leaves. Scale bars = 50 µm.

(c) *GSTU24* expression induced by 100 nM flg22 or mock (0.1% DMSO) at 2 hpi. *PP2AA3* was used as the internal control and data was normalized to the average gene expression in mock-treated Col-0. Different letters indicate statistically significant differences (p<0.05), which were determined by Two-way ANOVA with Tukey’s HSD test. Biological replicates: n=4.

**Table S1.** Primers used in qRT-PCR.

| **AGI number** | **Primer name** | **Primer sequence (5'-3')** | **Source** |
| --- | --- | --- | --- |
| AT1G13320 | PP2AA3_qRT_F | GGTTACAAGACAAGGTTCACTC | Huot et al., 2017 |
|  | PP2AA3_qRT_R | CATTCAGGACCAAACTCTTCAG |  |
| AT2G19190 | FRK1_qRT_F | CAGAAACAGCGCGAAACGA |  |
|  | FRK1_qRT_R | GGTCGGGCGGTCTGAAA |  |
| AT4G23550 | WRKY29_qRT_F | CTCCATACCCAAGGAGTTATTACAG | Cheng et al., 2013 |
|  | WRKY29_qRT_R | CGGGTTGGTAGTTCATGATTG |  |
| AT5G57220 | CYP81F2_qRT_F | GTGAAAGCACTAGGCGAAGC |  |
|  | CYP81F2_qRT_R | ATCCGTTCCAGCTAGCATCA |  |
| AT2G14610 | PR1_qRT_F | GGCTAACTACAACTACGCTG | Huot et al., 2017 |
|  | PR1_qRT_R | TCTCGTTCACATAATTCCCAC |  |
| AT1G74710 | ICS1_qRT_F | ACTTACTAACCAGTCCGAAAGACGA | Huot et al., 2017 |
|  | ICS1_qRT_R | ACAACAACTCTGTCACATATACCG |  |
| AT5G26920 | CBP60g_qRT_F | GATGACATGACCTCAAGCTG | Kong et al., 2016 |
|  | CBP60g_qRT_R | TTAACCTTACACCACCTGGC |  |
| AT1G17170 | GSTU24_qRT_F | AGACTTGGCCCGACAATAAC | Chaouch et al., 2010 |
|  | GSTU24_qRT_R | TCCTTCTCGCCGTAACATTC |  |

**MATERIALS AND METHODS**

**Plant and pathogen materials and growth conditions**

Wild-type and mutant lines of *Arabidopsis thaliana* used in the study are all from the ecotype Col-0. Mutants *hpr1-1* (SALK_067724) and *plgg1-1* (SALK_053469) were characterized previously (Timm *et al.*, 2008; Pick *et al.*, 2013). The previously characterized *rps2-101c* mutant (Mindrinos *et al.*, 1994) and *Pseudomonas syringae* pv. *tomato* strain *Pst* DC3000 (*avrPpt2*) were kindly provided by Dr. Yi-Ju Lu and Dr. Li Pai from the lab of Dr. Brad Day (Michigan State University). Peptide flg22 was obtained from PhytoTech Labs (www.phytolab.com). *Pst* DC3000 and *Pst* DC3000 (*avrPpt2*) were grown on Modiﬁed Luria-Bertani medium (LM, 10.0 g Bacto tryptone, 6.0 g Bacto yeast extract, 1.5 g K_2_HPO_4_, 0.6 g NaCl, 0.4 g MgSO_4_·7H_2_O per liter H_2_O) at 28-30 °C with Rif_50_ and Rif_50_/Kan_50_ respectively.

Arabidopsis seeds were directly sown in the soil in pots. After seed stratiﬁcation in the dark at 4 °C for 3 to 7 days, pots were moved to growth chambers with ~100 μmol m^-2^ s^-1^ white light, 21 °C, and 12 h/12 h light/dark cycle for plant growth.

**Pathogen infection assays**

Bacterial infiltration in Arabidopsis was performed using a previously published protocol (Yao *et al.*, 2013). Brieﬂy, leaves of 5-week-old Arabidopsis plants were syringe-infiltrated with a bacterial suspension [~1 × 10^5^ colony-forming units (CFU) ml^−1^ *Pst* DC3000 or ~ 1 × 10^6^ CFU ml^−1^ *Pst* DC3000 (*avrRpt2*) in 0.25 mM MgCl_2_ solution]. Plants were dried in the air in the growth chamber and covered with domes to keep high humidity. Two or three days after inoculation, i.e., 2 dpi for *Pst* DC3000 and 3 dpi for *Pst* DC3000 (*avrPpt2*), leaf tissue was collected and ground, and bacterial populations were determined by serial dilutions and colony counting under a stereo microscope.

For flg22 protection assays, plant leaves were syringe-inﬁltrated with 100 nM ﬂg22 or 0.1% DMSO (dimethyl sulfoxide, mock). After 22–24 h, plants in the growth chamber without domes were infiltrated again with a bacterial suspension [~ 1 × 10^6^ CFU ml^−1^ *Pst* DC3000 in 0.25 mM MgCl_2_ solution]. Bacterial populations were quantified at 2 dpi using the above-mentioned method.

**RNA extraction and qRT-PCR**

Leaves of 5-week-old plants were infiltrated with 100 nM flg22 or 0.1% DMSO. After ~2 h or ~24 h, leaf tissue was sampled and ground in liquid nitrogen. RNA was extracted using the NucleoSpin RNA Plant kit (MACHEREY-NAGEL), then reverse transcribed using the High Capacity cDNA Reverse Transcription Kit (Applied Biosystems). Fast SYBR Green Master Mix (Applied Biosystems) was used with a 7500 Fast Real-Time PCR System (Applied Biosystems) for real-time PCR. The primers used in qRT-PCR are listed in Table S1, with *PP2AA3* as the internal control gene. Data for each gene was normalized to its average gene expression in mock-treated Col-0.

**Apoplastic ROS burst assay**

As described previously (Zhang *et al.*, 2019), 4 mm leaf discs were taken from ~5-week-old plants and kept floating on water (adaxial side up) in 96-well plates overnight. Then water was removed and replaced with 100 μl immune-eliciting solution [34 μg ml^-1^ luminol (Sigma-Aldrich), 20 μg ml^-1^ horseradish peroxidase (Sigma-Aldrich) and 100 nM ﬂg22 (0.1% DMSO for mock) in water]. Luminescence was measured at 470 nm with a SpectraMax L microplate reader (Molecular Devices).

**H_2_DCFDA staining**

Leaf discs (4 mm) were taken from ~5-week-old plants and kept floating on water (abaxial side up) in 96-well plates overnight. Then water was removed and leaf discs were stained with 200-μl solution [100 μM H_2_DCFDA (Ambeed) and 0.02% Silwet L-77] for 30 min. Next, leaf discs were rinsed in water and observed with a Leica Stellaris 5 confocal microscope. Images were captured using a 63x oil objective and the following parameters: excitation at 488 nm and emission window of 500-560nm for DCF; and excitation at 405 nm and emission window of 600-750nm for chlorophyll autofluorescence. In the process, leaf discs were placed as long as possible in an environment with similar light intensity and photoperiod as those in the original growth chamber.

**RNA sequencing and data analysis**

Five-week-old plants were infiltrated with 100 nM flg22 or 0.1% DMSO. After ~2 h, leaf tissue was sampled and ground in liquid nitrogen. RNA was extracted using the NucleoSpin RNA Plant kit (MACHEREY-NAGEL), followed by additional DNA removal using the TURBO DNA-free Kit (Invitrogen).

RNA-seq was performed by the MSU Genomics Core, who prepared mRNA libraries with the Watchmaker Genomics mRNA Library Preparation Kit and pooled them together with samples from other researchers onto an Element Biosciences AVITI Cloudbreak Freestyle High Output 300 cycle flow cell. The sequencing yielded 2x150 bp paired-end reads with ~20M read pairs per sample.

Raw reads from RNA-seq were first trimmed with Trim Galore! v0.6.10 (<https://zenodo.org/records/7598955>). Then, STAR v2.7.11b (Dobin *et al.*, 2013) was used to align the filtered reads to the Arabidopsis reference genome TAIR10 (GCA_000001735.1) and the alignments were projected onto the transcriptome with the parameter --quantMode TranscriptomeSAM. Further, the reference transcriptome was generated with gffread v0.12.7 (Pertea and Pertea, 2020) and used for transcript quantification in Salmon v1.10.1 (Patro *et al.*, 2017) with --seqBias and --gcBias enabled.

DESeq2 v1.48.1 was used to perform differential expression analysis (Love *et al.*, 2014). Tximport v1.36.1 was used to import transcript abundances and construct a gene-level DESeqDataSet object from Salmon quant.sf files (Soneson *et al.*, 2016). Genes were filtered to only keep those with a count of at least 10 in 4 samples. Count data transformed by rlog (regularized logarithm) were used for PCA and heatmap visualization.

Gene ontology (GO) enrichment analysis (Biological Process category) was performed using the GO Term Enrichment Tool on the TAIR website (<https://v2.arabidopsis.org/tools/go_term_enrichment.jsp>).

For data visualization, PCA and GO enrichment plots were generated using ggplot2 v 4.0.0 (Wickham, 2016). Heatmaps were produced using pheatmap v1.0.13 (<https://github.com/raivokolde/pheatmap>), with genes (rows) clustered by hierarchical clustering using Euclidean distance and complete linkage.

**Statistics and plots**

Data analysis was performed using Microsoft Excel and Rstudio. One-way or Two-way ANOVA with Tukey’s HSD test was used for multi-comparisons. Each boxplot center line shows the median, and the lower and upper hinges correspond to the first and third quartiles. Whiskers extend from the hinges to the largest or smallest values that are no further than 1.5 times of the interquartile range. Data points are represented as individual dots.

**Supplementary References**

**Chaouch, S., Queval, G., Vanderauwera, S., Mhamdi, A., Vandorpe, M., Langlois-Meurinne, M., Breusegem, F. Van, Saindrenan, P. and Noctor, G.** (2010) Peroxisomal hydrogen peroxide is coupled to biotic defense responses by ISOCHORISMATE SYNTHASE1 in a daylength-related manner. *Plant Physiol.*, **153**, 1692–1705.

**Cheng, C., Gao, X., Feng, B., Sheen, J., Shan, L. and He, P.** (2013) Plant immune response to pathogens differs with changing temperatures. *Nat. Commun.*, **4**, 1–9.

**Dobin, A., Davis, C.A., Schlesinger, F., Drenkow, J., Zaleski, C., Jha, S., Batut, P., Chaisson, M. and Gingeras, T.R.** (2013) STAR: ultrafast universal RNA-seq aligner. *Bioinformatics*, **29**, 15–21.

**Huot, B., Castroverde, C.D.M., Velásquez, A.C., et al.** (2017) Dual impact of elevated temperature on plant defence and bacterial virulence in Arabidopsis. *Nat. Commun.*, **8**, 1–11. Available at: http://dx.doi.org/10.1038/s41467-017-01674-2.

**Jiang, X., Koenig, A.M., Walker, B.J. and Hu, J.** (2025) A cytosolic glyoxylate shunt complements the canonical photorespiratory pathway in Arabidopsis. *Nat. Commun.*, **16**, 4057.

**Kong, Q., Sun, T., Qu, N., et al.** (2016) Two redundant receptor-like cytoplasmic kinases function downstream of pattern recognition receptors to regulate activation of SA biosynthesis. *Plant Physiol.*, **171**, 1344–1354.

**Love, M.I., Huber, W. and Anders, S.** (2014) Moderated estimation of fold change and dispersion for RNA-seq data with DESeq2. *Genome Biol.*, **15**, 550.

**Mindrinos, M., Katagiri, F., Yu, G.-L. and Ausubel, F.M.** (1994) The *A. thaliana* disease resistance gene RPS2 encodes a protein containing a nucleotide-binding site and leucine-rich repeats. *Cell*, **78**, 1089–1099.

**Patro, R., Duggal, G., Love, M.I., Irizarry, R.A. and Kingsford, C.** (2017) Salmon provides fast and bias-aware quantification of transcript expression. *Nat. Methods*, **14**, 417–419.

**Pertea, G. and Pertea, M.** (2020) GFF Utilities: GffRead and GffCompare. *F1000Res.*, **9**, 304.

**Pick, T.R., Bräutigam, A., Schulz, M.A., Obata, T., Fernie, A.R. and Weber, A.P.M.** (2013) PLGG1, a plastidic glycolate glycerate transporter, is required for photorespiration and defines a unique class of metabolite transporters. *Proc. Natl. Acad. Sci. U. S. A.*, **110**, 3185–3190.

**Soneson, C., Love, M.I. and Robinson, M.D.** (2016) Differential analyses for RNA-seq: transcript-level estimates improve gene-level inferences. *F1000Research 2016 4:1521*, **4**, 1521.

**Timm, S., Nunes-Nesi, A., Pärnik, T., et al.** (2008) A cytosolic pathway for the conversion of hydroxypyruvate to glycerate during photorespiration in Arabidopsis. *Plant Cell*, **20**, 2848–2859.

**Wickham, H.** (2016) *ggplot2: Elegant graphics for data analysis*, Cham: Springer International Publishing.

**Yao, J., Withers, J. and He, S.Y.** (2013) Pseudomonas syringae infection assays in Arabidopsis. In A. Goossens and L. Pauwels, eds. *Jasmonate Signaling: Methods and Protocols*. Totowa, NJ: Humana Press, pp. 63–81.

**Zhang, L., Paasch, B.C., Chen, J., Day, B. and He, S.Y.** (2019) An important role of l-fucose biosynthesis and protein fucosylation genes in Arabidopsis immunity. *New Phytologist*, **222**, 981–994.
